# An Anisotropic Hydrogel by Programmable Ionic Crosslinking for Sequential Two-Stage Actuation under Single Stimulus

**DOI:** 10.3390/gels9040279

**Published:** 2023-03-29

**Authors:** Yanjing Zhang, Xingyu Cao, Yuyu Zhao, Huahuo Li, Shengwei Xiao, Zhangxin Chen, Guobo Huang, Ye Sun, Zhenzhong Liu, Zhicai He

**Affiliations:** 1 School of Pharmaceutical and Chemical Engineering, Taizhou University, Taizhou 318000, China; 2 State Key Laboratory of Marine Resource Utilization in South China Sea, Hainan University, Haikou 570228, China; 3 Research Institute of Zhejiang University-Taizhou, Taizhou 318000, China

**Keywords:** bi-layer hydrogel, two-stage actuation, pH response, programmable, single stimulus

## Abstract

As one of the most important anisotropic intelligent materials, bi-layer stimuli-responsive actuating hydrogels have proven their wide potential in soft robots, artificial muscles, biosensors, and drug delivery. However, they can commonly provide a simple one-actuating process under one external stimulus, which severely limits their further application. Herein, we have developed a new anisotropic hydrogel actuator by local ionic crosslinking on the poly(acrylic acid) (PAA) hydrogel layer of the bi-layer hydrogel for sequential two-stage bending under a single stimulus. Under pH = 13, ionic-crosslinked PAA networks undergo shrinking (-COO^−^/Fe^3+^ complexation) and swelling (water absorption) processes. As a combination of Fe^3+^ crosslinked PAA hydrogel (PAA@Fe^3+^) with non-swelling poly(3-(1-(4-vinylbenzyl)-1H-imidazol-3-ium-3-yl)propane-1-sulfonate) (PZ) hydrogel, the as-prepared PZ-PAA@Fe^3+^ bi-layer hydrogel exhibits distinct fast and large-amplitude bidirectional bending behavior. Such sequential two-stage actuation, including bending orientation, angle, and velocity, can be controlled by pH, temperature, hydrogel thickness, and Fe^3+^ concentration. Furthermore, hand-patterning Fe^3+^ to crosslink with PAA enables us to achieve various complex 2D and 3D shape transformations. Our work provides a new bi-layer hydrogel system that performs sequential two-stage bending without switching external stimuli, which will inspire the design of programmable and versatile hydrogel-based actuators.

## 1. Introduction

Nowadays, advances in big health industries and artificial intelligence have engendered the vigorous development of intelligent materials [1,2]. How to improve the intelligence degree of materials is one of the core issues in this field. Stimuli-responsive hydrogels [3,4,5], as a class of elastomers, have attracted more and more attention due to their great biocompatibility, soft-wet, and structural/composition designability. Many efforts have demonstrated their great potential in soft robots [6], biosensors [7,8], drug delivery [9], and so on [10]. Thanks to the distribution of a large number of identifiable groups (e.g., hydrophilic and/or dissociative groups) in the polymer networks, some impressive reversible changes in shape, phase state, and color of hydrogels happen when triggered by external environmental factors, such as pH [11], ion strength [12,13,14], temperature [15], light field [16], electric field [17], magnetic field [18], etc. Therefore, selecting an appropriate trigger mode (single or multi-stimuli) and the matched polymer types/structures are two keys to developing smart hydrogels that meet strict requirements.

In general, hydrogel materials are typically isotropic, which determines they only express uniform volume changes under external stimuli. This is far from the deformation of natural organisms, such as the Venus flytrap [19], mimosa [20], and fern sporangia [21], etc., in response to an emergency. Bi-layer hydrogel actuators integrate different or even contrary functions on two sides [22,23,24,25,26], enabling simple unidirectional or bidirectional bending. The shape transformation driving force arises from the local asymmetric volume change caused by an imbalance in internal stress, especially at the interface. On the basis of this principle, various bi-layer hydrogels have been developed to simulate some responsive actions for grasping, scrolling, and bouncing. For example, Peng et al. [27] fabricated a bi-layer hydrogel by in situ polymerizing NIPAM/CNF precursors onto another PAM-AA/CNF surface. By switching the temperature from 45 °C to 25 °C, the bi-layer exhibited bending and recovery abilities. Ma et al. [28] combined photothermal responsive PPy-P(NIPAM-ABP) hydrogel with unresponsive PEGDA-CNF to obtain an active/passive bi-layer hydrogel through two-step polymerization. This bi-layer hydrogel could simulate the long-distance crawl of starfish by repeating laser “irradiation ↔ cancellation”. Our previous work reported a one-step method for preparing bi-layer hydrogel by mixing zwitterionic and N-isopropyl acrylamide (NIPAM) monomers [29,30]. The bi-layer structure resulted from the self-separation of two monomers during polymerization. Due to the opposite salt and temperature response of the two layers, the bi-layer hydrogel showed bidirectional bending under the “water ↔ salt” or “hot water ↔ cold water”. However, both these unidirectional and bidirectional bending behaviors are only achievable under different environments or stimuli, which greatly limits their practical application.

To overcome this issue, recently, some interesting heterogeneous hydrogels have been fabricated to achieve multi-directional bending in response to a single external stimulus. For example, Sui et al. [31] constructed a gradient CS/SA hydrogel by diffusing low molecular weight chitosan into high-molecular-weight sodium alginate. Due to the competitive effect between complexed and noncomplexed segments of polyelectrolyte, this hydrogel showed a distinct sequential double-folding behavior in response to temperature, ionic strength, and pH. In addition, Ye et al. [32] prepared a heterogeneous PNIPAM/MMT hydrogel with a double gradient in structure and composition under gravity. Under 50 °C, this gradient composite hydrogel could achieve a sequential bidirectional bending due to the different shrinkage between the top and bottom sides. Although these attempts provide a good reference for the design of new heterogeneous hydrogels in the future, their defects are visible, that is, non-uniform penetration or precipitation can occur unpredictably during the preparation of large-scale samples. Therefore, it is urgent to develop a simple and convenient strategy for preparing large-scale heterogeneous hydrogels.

**Scheme 1:**
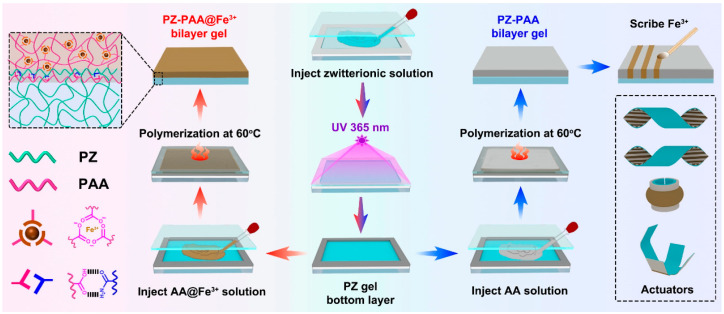
Schematic illustration of the fabrication process of PZ-PAA@Fe^3+^ bi-layer hydrogel and the programmable heterogeneous hydrogel actuators.

Herein, we proposed a new PZ-PAA@Fe^3+^ bi-layer hydrogel capable of achieving tunable, fast, and sequential two-stage bending under a single stimulus (Scheme 1). The different swelling properties of two sides of bi-layer hydrogels are expected to endow tunable actuation behavior. Most importantly, when triggered by pH = 13, this bi-layer hydrogel showed a fast and controllable two-stage bending. Two factors are responsible for this unique bending behavior: (1) under pH = 13, the counter ions (0.1 mol/L Na^+^/OH^−^) increase the complexation density of PAA@Fe^3+^ at first (shrinking), and then the ionized -COOH (-COO^−^) cause the water absorption of PAA networks (swelling); (2) the counter ions of 0.1 mol/L are not sufficient to induce the significant volume changes of PZ in a short time. Moreover, the volume changes of both layers showed a temperature dependence, which cooperatively induced a faster shape deformation of the bi-layer hydrogel. The bending orientation, angle, and velocity also could be regulated by the Fe^3+^ concentration and the thickness of the hydrogel. By locally crosslinking Fe^3+^ with PAA, a series of programmable heterogeneous hydrogels could be customized and further achieved various complex 2D and 3D shape transformations. Our strategy to achieve sequential two-stage bending under a single stimulus can be expanded to other hydrogels in combination with PAA@Fe^3+^ hydrogel, and bring new insights into the design of programmable and versatile smart biomimetic actuators.

## 2. Results and Discussion

### 2.1. Fabrication and Swelling-Shrinking Properties of PZ-PAA@Fe^3+^ Bi-Layer Hydrogel

The PZ-PAA@Fe^3+^ bi-layer hydrogel was fabricated using a typical two-step strategy, as shown in Scheme 1. First, the zwitterionic PZ hydrogel layer was obtained through UV-photopolymerization, the core of which was zwitterionic polymer segments. Then, the AA and FeCl_3_ precursor solutions were injected into the PZ hydrogel under ice-water protection. During the subsequent thermal polymerization, the slow diffusion of the AA@Fe^3+^ precursor solutions contributed to interpenetrating networks and provided tough interfacial strength. The introduction of Fe^3+^ could coordinate with -COO^−^, which on the one hand helps to improve the mechanical properties of the hydrogel, on the other hand, the complexation of -COO^−^/Fe^3+^ can also change the swelling of PAA hydrogel. In parallel, the bi-layer hydrogel was composed of PZ and PAA without Fe^3+^. Then, Fe^3+^ was introduced into the PAA networks by hand-painting, resulting in various heterogeneous hydrogels with different patterns. Therefore, it is reasonable to believe that various 2D and 3D shape transformations of the hydrogel can be achieved due to the difference in local swelling and shrinking.

**Figure 1:**
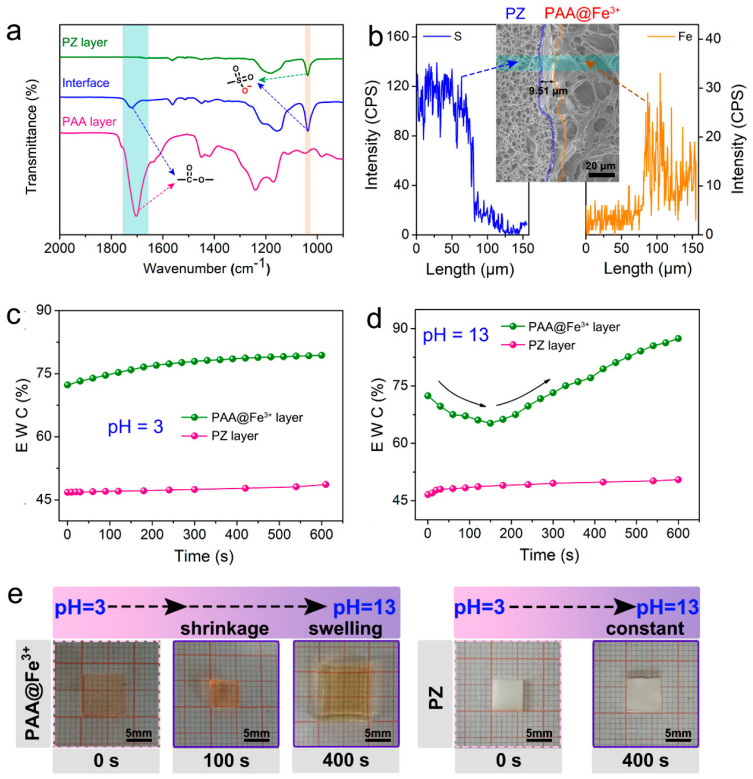
(**a**) Comparison of FTIR spectra of PZPAA@Fe^3+^ bi-hydrogel in different area. (**b**) SEM image and EDS measurement of bi-layer hydrogel. (**c**,**d**) Equilibrium water contents (EWC) and (**e**) swelling/shrinking behavior of PZ hydrogel and PAA@Fe^3+^ hydrogel from pH = 3 to pH = 13.

The as-prepared bi-layer structure was confirmed by Fourier-transform infrared spectroscopy (FTIR) and X-ray photoelectron spectroscopy (XPS) (Figure 1a and Figure S1). The peak at 1038 cm^−1^ was assigned to the stretching vibration of -SO_3_^−^ from PZ hydrogel; the strong absorption band at 1724 cm^−1^ was assigned to -COO^−^ from PAA hydrogel. Meanwhile, both -SO_3_^−^ and -COO^−^ groups were observed at the intersection, demonstrating that the double networks were formed at the interface between PZ hydrogel and PAA@Fe^3+^ hydrogel. The scanning electron microscopy (SEM) image in Figure 1b clearly showed two different pore structures, where the small and compact porous structure came from the PZ layer versus the large porous structure from the PAA@Fe^3+^ layer. In addition, the elemental scanning (EDS) results of the sulfur element and iron element with unique attribution both showed obvious step shapes, which further indicated the bi-layer structure and interpenetrate networks at the interface.

Usually, the driving force for the shape transformation of the bi-layer hydrogel comes from the asymmetric swelling-shrinking properties. So, we investigated the swelling behaviors of PZ and PAA@Fe^3+^ hydrogels in different buffer solutions. As shown in Figure 1c,d, PZ hydrogel showed nearly no obvious swelling both in pH = 3 and pH = 13, indicating that the zwitterionic hydrogels used in this work were not sensitive to pH. Considering the “anti-polyelectrolyte effect” of zwitterionic polymers [33], we further studied the influence of ionic concentration on the swelling behavior of zwitterionic hydrogel in Figure S2. Clearly, low ionic concentration (0.1 mol/L NaCl) could not improve the equilibrium water content (EWC) of PZ hydrogel effectively, while the “anti-polyelectrolyte effect” was gradually revealed only when the ionic concentration increased to 0.5 mol/L. The results further demonstrate that the counter ions in pH = 3 and 13 (10^−11^ mol/L and 0.1 mol/L of Na+/OH^−^) cannot promote the swelling of PZ hydrogel. For PAA@Fe^3+^ hydrogel, EWC hardly changed over time under pH = 3. In contrast, the hydrogel experienced an unexpecting process of first deswelling and then swelling (72%→65%→87%) under pH = 13. This phenomenon may be attributed to the complexation of -COO^−^/Fe^3+^ and the ionization of -COOH. Firstly, the counter ions (Na^+^/OH^−^) penetrate into PAA networks rapidly and accelerate the coordinating between Fe^3+^ and -COO^−^, giving rise to the increase in complexation density; secondly, with the increase of the ionization of -COOH, the water absorption of -COO^−^·Na^+^ gradually dominates, leading to the swelling of PAA@Fe^3+^ hydrogels. The swelling behavior in Figure S3 confirmed that the swelling ratio of -COO^−^/Fe^3+^ hydrogel was hardly affected by low ionic concentrations (<0.5 mol/L). It is noted that PAA@Fe^3+^ hydrogel does not duplicate this strange swelling behavior from pH = 7 to 12. We believe that this sequential shrinking-swelling behavior will provide a unique driving force for actuators.

### 2.2. pH and Thermal Induced Sequential Two-Stage Bending of PZ-PAA@Fe^3+^ Bi-Layer Hydrogel

**Figure 2:**
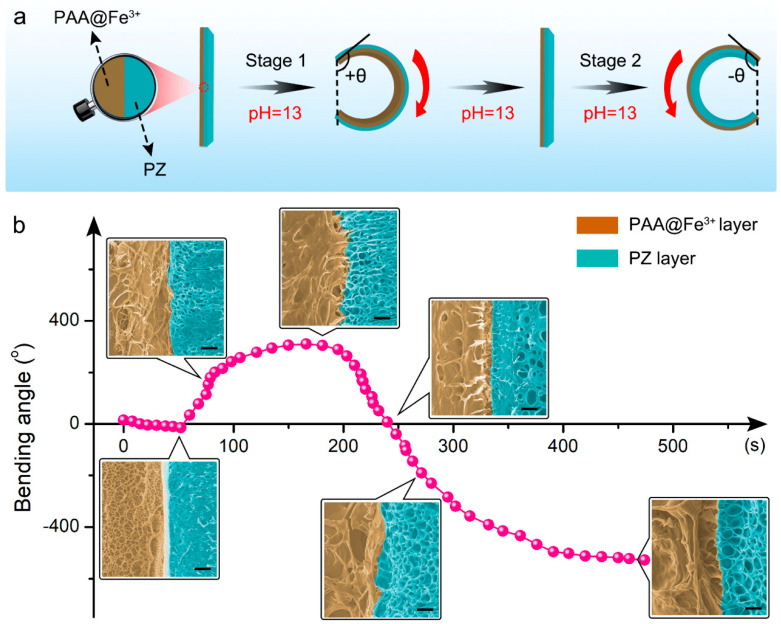
(**a**) Schematic illustration of the shape deformation of PZ-PAA@Fe^3+^ bi-layer hydrogel strip. The bi-layer hydrogel at pH = 13 first bent toward the PAA@Fe^3+^ side (+), then became straight, and finally bent toward the PZ side (−). (**b**) Bending deformation of PZ-PAA@Fe^3+^ bi-layer hydrogel at pH = 13 and SEM images of the cross sections at different stages. All scale bars are 50 μm.

As we hypothesized, the different swelling properties of PZ and PAA@Fe^3+^ hydrogels drive their bi-layer strip to bend in a complex, interesting, but controlled way. Especially in pH = 13, the bi-layer strip presented a sequential two-stage deformation behavior, in which the straight bi-layer strip bent toward the PAA@Fe^3+^ side at first, then unbent and rebent toward the PZ side (Figure 2a). To better quantify the bending behavior, the bending angle is determined between the original point tangent and the original point-end edge vector. Accordingly, a positive bending angle is defined as the bi-layer strip curve clockwise (bending toward the PAA@Fe^3+^ side), while a negative bending angle is vice versa (bending toward the PZ side). On this basis, we obtained the bending kinetic curve of the PZ-PAA@Fe^3+^ bi-layer hydrogel in response to a pH change from pH = 3 to pH = 13, as shown in Figure 2b. Clearly, the bi-layer hydrogel strip underwent 2 stages, from initial negative bending and temporary positive bending to final negative bending within 400 s.

In addition, the microstructure of the bi-layer hydrogel at different bending stages was recorded by SEM to further explore its bending mechanism (insert photos in Figure 2b). It was observed that the bi-layer hydrogel bent toward the PZ side slightly in pH = 3, taking ~60 s to reach −14° due to the stiffness of PZ hydrogel and higher swelling of PAA@Fe^3+^ hydrogel (Figure 1c). Though once submerged in pH = 13, as described above, the counter ions both quickly penetrated the PAA and PZ networks, resulting in the shrinking of the PAA layer and the slight swelling of the PZ layer. Therefore, the synergistic effect of the opposite swelling behavior caused the bi-layer hydrogel strip to bend spontaneously from a negative (−14°) to a positive bending angle (34.4°) in 8 s. The bending angle continued to increase until 310.5° at 166 s, indicating that the complexation density of -COO^−^/Fe^3+^ reached the maximum. However, there was still a lot of -COO^−^ capturing water, causing the PAA@Fe^3+^ to swell even higher than that of PZ. As a result, the curved bi-layer hydrogel unfolded gradually and bent in reverse again.

**Figure 3:**
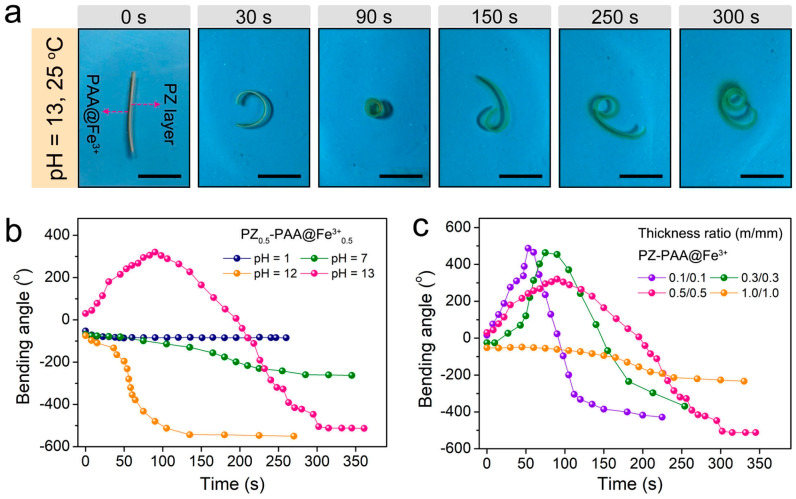
(**a**) Optical photos of bending process of PZ-PAA@Fe^3+^ bi-layer hydrogel strip in pH = 13 at 25 °C. Bending kinetics of bi-layer hydrogel strips affected by (**b**) different pH and (**c**) different thickness ratios of PZ-PAA@Fe^3+^. All scale bars are 10 mm.

The exploration of the bending mechanism of the PZ-PAA@Fe^3+^ bi-layer hydrogel shows that pH greatly affects the bending property. Akin to the typical example shown in Figure 3a, the whole bending process of a bi-layer hydrogel sample under pH = 13 was recorded. Overall, the PZ-PAA@Fe^3+^ bi-layer hydrogel underwent two stages of “positive bending → unfolding and reverse bending”. Due to the shrinking of the PAA layer and slight swelling of the PZ layer, the bi-layer hydrogel quickly bent toward the PAA@Fe^3+^ side, that is, reached the maximum positive bending degree of 320.5° within 90 s. Subsequently, the bi-layer hydrogel gradually unfolded because of the swelling of the PAA@Fe^3+^ layer. The continuous swelling induced the sample reverse bending to a negative degree of −443° in the next 210 s. However, this bi-layer hydrogel exhibited only unidirectional deformation in pH from 7 to 12 in Figure 3b. The reason for this phenomenon might be that the PAA networks’ shrinking from the complexation of PAA@Fe^3+^ was weaker than the swelling process from the ionization of -COOH under low counter ions. The hydrogel sample hardly bent under pH = 1, which was consistent with the swelling results in Figure 1c.

To understand the effect of bi-layer hydrogel thickness on bending behavior, 4 bi-layer hydrogels with different thicknesses of 0.2, 0.6, 1.0, and 2.0 mm were prepared. Herein, in order to eliminate the errors, the thickness ratio on both sides of the hydrogel was controlled as 1:1. As shown in Figure 3c, clearly, the 0.2 mm thickness bi-layer hydrogel (0.1/0.1) showed the highest sensitivity to pH. The time of bidirectional bending in pH = 13 for bi-layer hydrogels with a thickness of 0.2 (0.1/0.1), 0.6 (0.3/0.3), and 1.0 mm (0.5/0.5) were ~100 s, ~200 s, and ~300 s, respectively. However, the hydrogel with a thickness of 2.0 mm (1.0/1.0) only bent toward the PZ side because of the increasing modulus of both sides. 

**Figure 4:**
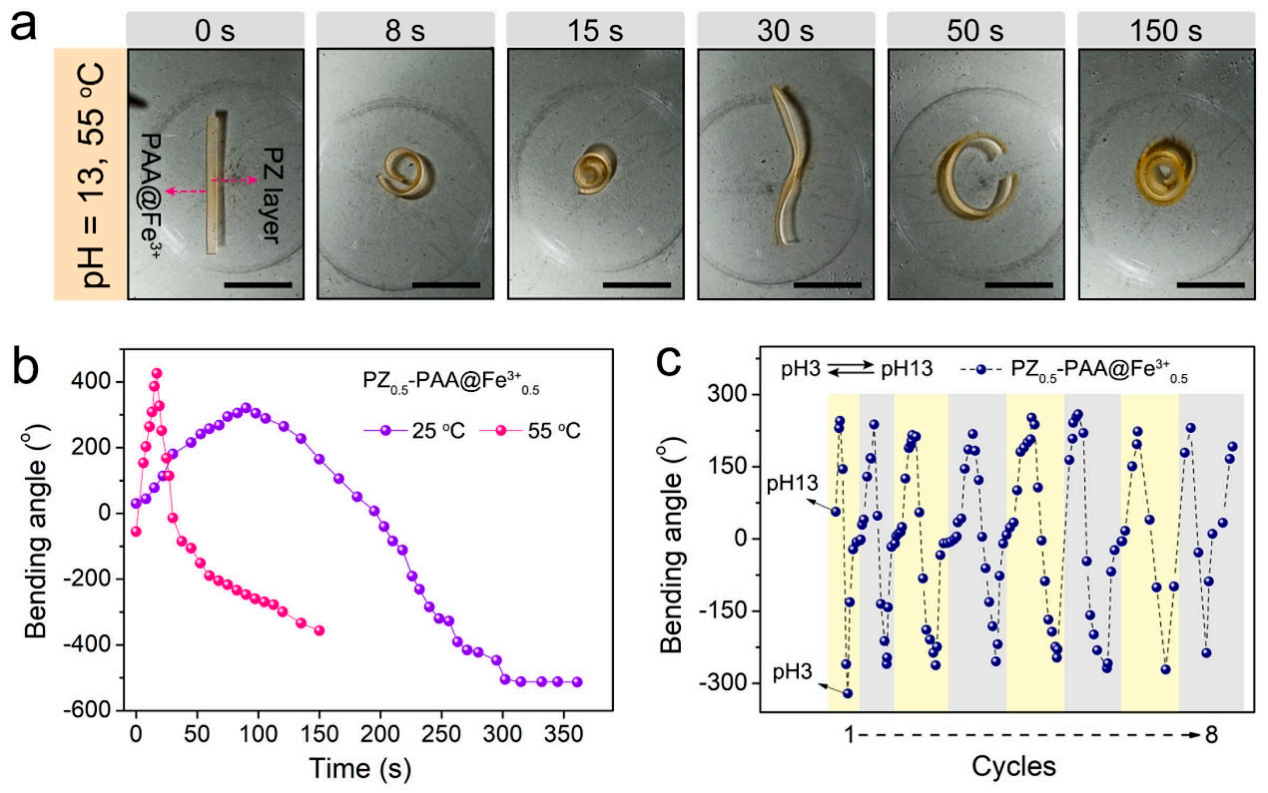
(**a**) Optical photos of bending process of PZ-PAA@Fe^3+^ bi-layer hydrogel strip in pH = 13 at 55 °C. (**b**) Bending kinetics of bi-layer hydrogel strips affected by temperature. (**c**) Reversible bending behavior of the bi-layer hydrogel between pH = 3 and pH = 13. All scale bars are 10 mm. All scale bars are 10 mm.

It is well known that the zwitterionic hydrogel shows thermo-responsive properties, usually shrinks at temperatures < upper critical soluble temperatures (UCST), and swells at temperatures >UCST [14,29]. We believe that the thermo-response of the zwitterionic hydrogel can greatly affect the bending behavior of PZ-PAA@Fe^3+^ bi-layer hydrogel. As shown in Figure S4, PZ hydrogel exhibited slight swelling from ~46% to ~48% within 200 s under pH = 13 at 25 °C while significantly swelling from ~47% to ~54% within 200 s at 55 °C. More importantly, the increase of alkali liquors temperature also accelerated the volume shrinking and swelling of PAA@Fe^3+^ hydrogel, indicating that hot alkali liquors promoted the rate of -COO^−^/Fe^3+^ complexation and -COO^−^·Na^+^ water absorption (Figure S5). Taken together, the changes in the volume of PZ and PAA@Fe^3+^ hydrogels induced a faster bending process of a bi-layer hydrogel strip. Compared to cool alkali liquors at 25 °C, PZ-PAA@Fe^3+^ bi-layer hydrogel strip only took 150 s to achieve the sequential two-stage bending in hot alkali liquors at 55 °C (Figure 4a). As shown in Figure 4b, the bi-layer hydrogel strip showed a positive bending rate of ~28.3 °/s at 25 °C, versus ~3.2 °/s at 55 °C. Such rapid bending rate is attributed to the synergistic effect of the swelling of the PZ layer and the shrinking of the PAA@Fe^3+^ layer in hot alkali liquors. Subsequently, the thermo-induced swelling of the PZ layer continues to accelerate the unfolding (~33.8 °/s) and reverse bending (~2.8 °/s) of the bi-layer hydrogel strip. 

The different swelling behaviors of PAA@Fe^3+^ hydrogel in pH = 3 and 13 indicated that PAA networks could be switched between ionization and protonation. Therefore, the PZ-PAA@Fe^3+^ bi-layer hydrogel could be endowed with an excellent repeatable and reversible bending process in and out of acidic and alkaline conditions (Figure 4c). The bi-layer hydrogel strip possessed the initial two-step bending of ~250° and ~−320° in pH = 13. When switching to the pH = 3 condition, the bi-layer rapidly unfolded into a straight state. By repeatedly switching stimuli between pH = 3 and pH = 13, the bi-layer hydrogel strip could be repeated at least 8 times without obvious attenuation. What we should note is that such repeat numbers may be limited by the incomplete recoverable complexation.

### 2.3. Mechanical Properties of the Bi-Layer Hydrogel

**Figure 5:**
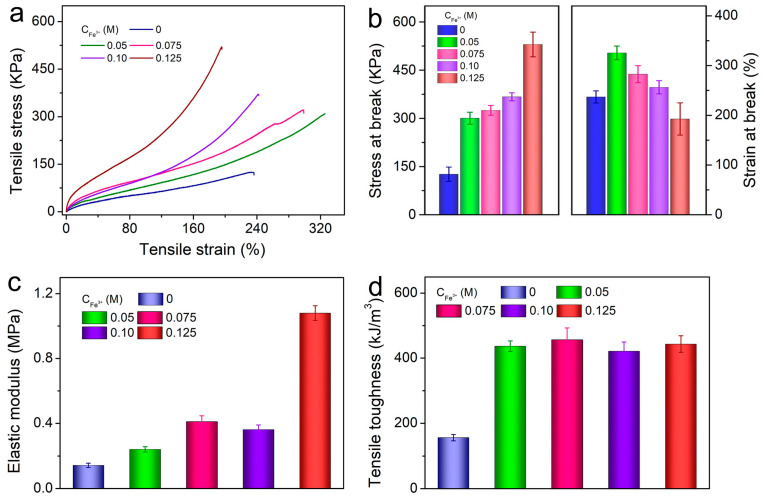
(**a**) Tensile stress-strain curves of PZ-PAA bi-layer hydrogels coordinating at different Fe^3+^ concentrations. (**b**) Stress and strain at break, (**c**) elastic modulus, and (**d**) tensile toughness of PZ-PAA@Fe^3+^ bi-layer hydrogel.

The complexation of -COO^−^/Fe^3+^ not only plays a vital role in the actuation of PZ-PAA@Fe^3+^ bi-layer hydrogel but also affects their mechanical properties. Thus, five bi-layer hydrogels coordinating with different Fe^3+^ concentrations were prepared, and their mechanical properties were further quantified by a tensile apparatus (Figure 5a). Both tensile stress and strain of PZ-PAA bi-layer hydrogel without coordinating with Fe^3+^ were quite low (~124 KPa, ~235%), while the mechanical parameters were notably increased after introducing Fe^3+^. In Figure 5b,c, when Fe^3+^ concentration increased from 0.05 to 0.125 M, the tensile stress and elastic modulus were improved gradually (~309, ~321, ~370, and ~519 KPa of stress, and ~0.24, ~0.14, ~0.36, and ~1.08 MPa of elastic modulus). However, the tensile strain decreased from ~326%, ~298%, and ~242% to ~196%, respectively. The superior mechanical properties of the PZ-PAA@Fe^3+^ bi-layer hydrogel are attributed to the increase in the complexation density of -COO^−^/Fe^3+^. In addition, as shown in Figure 5d, the work required for the break of the bi-layer hydrogel strip coordinated with Fe^3+^ was 436 ± 16.2 kJ/m^3^, which was nearly 4 times that of the bi-layer hydrogel strip without coordinating with Fe^3+^ (126.6 ± 9 kJ/m^3^). The results demonstrated the tensile toughness of bi-layer hydrogels was improved effectively after complexation, which provided strong support for their driving applications. 

Figure S6 showed the dependence of bending behavior on Fe^3+^ concentration in pH = 13. Interestingly, PZ-PAA without coordinating with Fe^3+^ showed a similar curvature to PZ-PAA@Fe^3+^ bi-layer strip. Clearly, the PZ-PAA bi-layer hydrogel strip also experienced firstly positive bending (57°) and secondly negative bending (−96.9°) process. In this case, the deformation only depends on the swelling competition between PZ and PAA layers. At first, although both PZ and PAA layers are swollen, the fast water absorption and softness of the PAA hydrogel cannot actuate the slow swelling and stiffness of the PZ hydrogel, so the bi-layer hydrogel strip bends toward the PAA side. Then, the driving force of the PAA layer gradually dominates the deformation after the PZ hydrogel is softened by counter ions, leading the hydrogel to achieve reverse bending. We should note that both the positive and negative bending of the PZ-PAA bi-layer hydrogel is smaller than that of the PZ-PAA@Fe^3+^ bi-layer hydrogel. For PZ-PAA@Fe^3+^ bi-layer hydrogel, relatively higher Fe^3+^ concentrations (0.075 M) result in a faster bending rate than 0.05 M Fe^3+^. Miraculously, as the Fe^3+^ concentration increased to 0.10 M, the first stage of the positive bending process disappeared, which provided the foundation for assembling bionic materials capable of 3D deformation.

### 2.4. Programmable Shape Transformations by Locally Fe^3+^ Coordinating Patter and Biomimetic Applications

**Figure 6:**
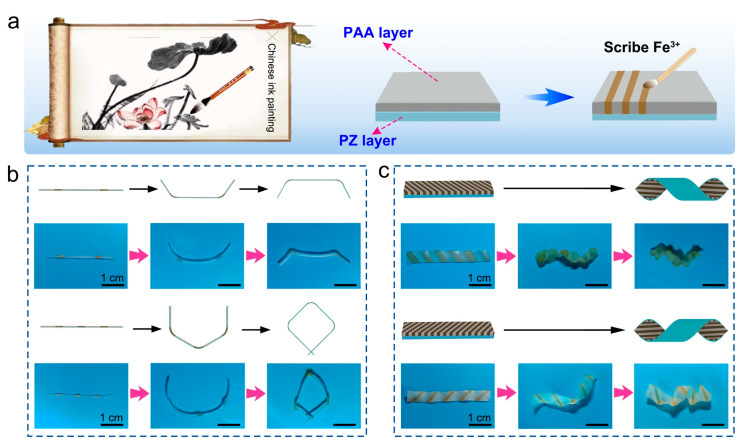
Fabrication of PZ-PAA@Fe^3+^ heterogeneous hydrogel actuators through painting Fe^3+^ solutions onto PZ-PAA surface (PAA side) and their 2D and 3D deformation in response to pH = 13. (**a**) Schematic illustration of the locally Fe^3+^ Coordinated PZ-PAA@Fe^3+^ heterogeneous hydrogel. Controllable actuation of (**b**) dot- and (**c**) stripe-patterned hydrogels.

On the basis of the bending behavior of PZ-PAA@Fe^3+^ hydrogel, we have found that a typical bi-layer hydrogel strip can only change shape from 1D to 2D due to the uniform swelling/shrinking on one or both sides. It is conceivable that if another heterogeneous structure is designed on one side of the bi-layer hydrogel, this bi-layer hydrogel will achieve complex deformation from 1D to 3D under stimulus [34]. Inspired by Chinese ink painting, we used a swab soaked with Fe^3+^ solutions to hand-paint a series of customizable patterns on the surface of PAA hydrogel (Figure 6a). During the permeation, local PAA polymer chains were constrained by Fe^3+^ along the thickness orientation, thus leading to interesting but controllable complex deformations. For example, three- and four-segment bi-layer hydrogel strips were prepared through dot-permeating (Figure 6b). Given that the coordinate -COO^−^/Fe^3+^ area had a faster deformation rate, the nodes promoted the hydrogel strips’ presentation of site-specific folding, undergoing the 2D deformation from “∪” to “∩” and “◇”, respectively. In another interesting design, we expanded the size of the dot and obtained a stripe-patterned bi-layer hydrogel sheet (Figure 6c). Endowing a stimulus, the mismatch in swelling between the stripes leads to an interesting deformation from 2D to 3D. Typically, when the angle of the strip was 135°, the bi-layer hydrogel sheet self-twisted into a right-handed helix shape in pH = 13. In parallel, the bi-layer hydrogel sheet with a strip angle at 45° self-twisted into a left-handed helix shape. Obviously, the helical density and twisting orientation can be easily tuned by hand-painting the different sizes and angles of strips.

**Figure 7:**
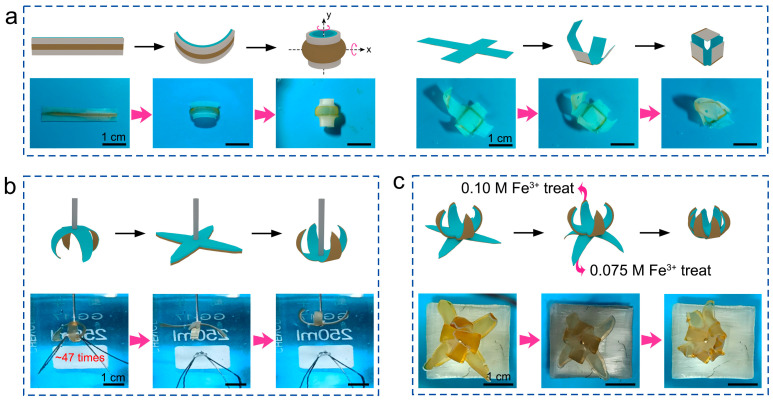
(**a**) Asymmetric deformation from planar sheet to lantern and folded box. (**b**) “Capturing-releasing” process of a plastic block by a cross-shaped hydrogel in pH = 13. (**c**) Multi-step deformation of the biomimetic hydrogel flower (the hydrogel petals are treated with 0.075 and 0.10 M Fe^3+^, respectively).

Further widening the width of PAA@Fe^3+^ hydrogel into a rectangular band across PAA hydrogel (Figure 7a), the hydrogel sheet showed unexpected synchronous double-folding deformation along the distinct axis in pH = 13. On the one hand, the bi-layer hydrogel bent around the y-axis into a closed loop under stimulus; on the other hand, PAA@Fe^3+^ hydrogel bent around the x-axis because of the stress constraints in the interconnecting region between PAA and PAA@Fe^3+^. In addition, the deformation from the 2D plane to the 3D cube can be presented by combining a bi-layer hydrogel tailored into a special shape with locally Fe^3+^-constrained PAA hydrogels.

In order to demonstrate that the PZ-PAA@Fe^3+^ bi-layer hydrogel with continuous deformation property can be applied, we designed a four-arm gripper, as shown in Figure 7b. The hydrogel gripper initially bent downward to grasp the targeted object quickly in pH = 13 due to the cooperative driving force from the swelling of the PZ layer and the shrinking of the PAA@Fe^3+^ layer. The gripper then gradually unfolded and released the object. Definitely, transferring the targeted object can also be implemented during the folding-unfolding process. Interestingly, due to the relative stiffness of the PZ layer under pH = 13, such a biomimetic gripper could grasp objects up to ~47 times the weight of dried hydrogel at the first stage. However, as the increased swelling of both PZ and PAA@Fe^3+^ hydrogels, the driving force would decrease in the second stage. This result is consistent with the bending kinetics of the PZ-PAAFe^3+^ bi-layer hydrogel in Figure 2, which is a hydrogel showing rapid bending in the first stage but slow bending in the second stage. Quantifying the actuation force of the bi-layer strips will provide theoretical support for the design and applications of novel actuators. Noteworthy, one of the external stimuli in this work is a strong base; thus, this artificial soft actuator shows great competitiveness in some extreme conditions. Moreover, according to Figure S6, both the bending rate and orientation of the PZ-PAA@Fe^3+^ bi-layer hydrogel are highly dependent on the Fe^3+^ concentration. Therefore, we prepared an interesting biomimetic hydrogel flower, whose petals were treated with different concentrations of Fe^3+^. As shown in Figure 7c, the inner and outer petals were PZ-PAA@ (0.10 M) Fe^3+^ and PZ-PAA@ (0.075 M) Fe^3+^, respectively. Upon stimulating alkali liquor, the inner petals showed continuous introversion, while the outer petals showed blooming-closing changes.

## 3. Conclusions

In summary, we reported a pH and thermal-responsive bi-layer hydrogel, which was composed of the PZ layer and PAA@Fe^3+^ layers. The coordination between -COO^−^ and Fe^3+^ caused a unique sequential “shrinking → swelling” process of PAA@Fe^3+^ hydrogels in pH = 13. Thus, the PZ-PAA@Fe^3+^ bi-layer hydrogel presented a distinct two-stage curvature under the stimulus of pH = 13. Both the bending orientation and rate could be regulated by changing pH, Fe^3+^ concentration, temperature, and hydrogel thickness. Inspired by Chinese ink painting, in which inks can be imprinted into penetrable Xuan-paper, we drew patterns on PZ-PAA hydrogel (PAA side) using a swab soaked with Fe^3+^ solutions, resulting in a series of patterned PZ-PAA@Fe^3+^ heterogeneous hydrogels. Due to the local complexation of PAA@Fe^3+^, various 2D and 3D shape transformations with localized bending and chiral twisting were achieved. This simple hand-painting strategy enables the heterogeneous hydrogels to mimic natural organisms effectively. The results of this work not only present a novel multi-responsive bi-layer hydrogel actuator system but also provide a philosophy for designing complex programmable heterogeneous materials.

## 4. Materials and Methods

### 4.1. Materials

Zwitterionic monomer was synthesized according to our previous report [35,36]. Acrylamide (AM, 96%), Acrylic acid (AA, stabilized with MEHQ, 99.0%), N,N′-methylene-bis-acrylamide (MBAA), 2-hydroxy-4′-(2-hydroxyethoxy)-2-methylpropiophenone (photo-initiator, 98%), Ammonium persulfate (APS), and Iron (III) chloride hexahydrate (FeCl_3_·6H_2_O, 99%) were purchased from Aladdin Ltd. (Shanghai, China). All other chemicals and solvents were commercially obtained at extra-pure grade and were used as received. Water used in these experiments was purified by a Millipore water purification system with a minimum resistivity of 18.0 MΩ cm.

### 4.2. Bi-Layer Hydrogel Preparation

PZ-PAA@Fe^3+^ bi-layer hydrogel was fabricated by a two-step method. Firstly, zwitterionic precursors were prepared by dissolving 3-(1-(4-vinylbenzyl)-1H-imidazol-3-ium-3-yl) propane-1-sulfonate (zwitterionic monomers, 0.0326 mol) in water with MBAA (crosslinker, 1.9 × 10^−3^ mol), APS (thermos-initiator, 2.2 × 10^−4^ mol), and 2-hydroxy-4′-(2-hydroxyethoxy)-2-methylpropiophenone (photo-initiator, 2.7 × 10^−4^ mol). In parallel, PAA precursors were prepared by mixing acrylic acid (0.14 mol), MBAA (3.2 × 10^−4^ mol), APS (2.0 × 10^−4^ mol), and water. Both zwitterionic precursors and PAA precursors were stirred under the protection of ice bath for 30 min to form homogeneous solutions and then purged with N_2_ for 15 min to remove the oxygen. Next, zwitterionic precursors were first gently injected into a self-made glass mold, which was separated by a certain thickness of silicone spacer. The polymerization was carried out under UV light (365 nm) at room temperature for 3 h and obtained PZ hydrogel. Before polymerization, a certain concentration of Fe^3+^ (0 M, 0.05M, 0.075 M, 0.1 M, 0.125 M) was added into PAA precursors to form different AA@Fe^3+^ solutions and then injected into the PZ hydrogel. Subsequently, the polymerization was carried out under 60 °C for 5 h. After polymerization, the resultant PZ-PAA@Fe3+ bi-layer hydrogel was immersed in pure water to remove unreacted monomers and was cut into 20 × 1.5 mm^2^ (length × width) for bending behavior measurements. The thickness of the hydrogel layer was controlled by changing the total thickness from 0.2 to 2.0 mm while fixing the ratio thickness of zwitterionic layer to PAA layer at 1:1.

### 4.3. Patterned Heterogeneous Hydrogel Preparation

First, zwitterionic and PAA precursors were prepared akin to 4.2. Then, zwitterionic precursors were gently injected into a self-made glass mold and polymerized under UV light (365 nm) at room temperature for 3 h. After polymerization, a PZ hydrogel was obtained. Subsequently, AA precursors were injected into the PZ hydrogel and polymerized at 60 °C for 5 h. As a result, a PZ-PAA bi-layer hydrogel was prepared. Finally, a certain concentration of FeCl_3_ solutions (0.075 M) was prepared using a volumetric flask. In addition, a swab was dipped into FeCl_3_ solutions and painted on the surface of bi-layer hydrogel (PAA layer) to form various heterogeneous hydrogels with local crosslinked PAA. The post-treatment of the hydrogel was similar to that of the bi-layer hydrogels. Furthermore, a biomimetic hydrogel flower was prepared by integrating 0.075 M Fe^3+^ and 0.10 M Fe^3+^ treated PZ-PAA bi-layer hydrogels.

### 4.4. Characterization of Hydrogels

All hydrogel samples were freeze-dried for the next measurements. The chemical structure of bi-layer hydrogel was characterized by Fourier-transform infrared spectroscopic (FT-IR, Nicolet 5700, Thermo Nicolet, Waltham, MA, USA) with resolution at 4 cm^−1^ and 32 scans. The freeze-dried hydrogel was brittle-fractured in liquid nitrogen and the cross section was observed with a scanning electron microscope (SEM, HITACHI S-4800, HITACHI, Tokyo, Japan). The specific element of hydrogel was measured through an X-ray photoelectron spectroscopy (XPS, Thermo Scientific Nexsa, Thermo Fisher Scientific, Waltham, MA, USA). Tensile measurements were performed with a tensile tester (Instron MOD EL5567, MA) with a 100 N load cell and a speed of 10 mm min-1 at room temperature. The tested hydrogel samples were cut into a dumbbell shape (15 mm × 2 mm× 1 mm, length × width × thickness). The bending and 2D/3D transformation of the hydrogels were recorded by a digital camera, and the bending angles were calculated using protractor software.

### 4.5. Swelling Behaviors of Hydrogels

The equilibrium water content (EWC, %) of the PZ hydrogel and PAA@Fe^3+^ hydrogel was determined as weight of the wet hydrogel relative to corresponding dry hydrogel. Each original hydrogel was punched into 8 mm disc with 3 parallel specimens and weighed as m_d_. The specimens were immersed in water for 3 days and weighed as m_w_.

EWC = (mw − md)/mw × 100%

## Data Availability

The data in this work are available from the corresponding author upon reasonable request.

## Supplementary Materials

The following supporting information can be downloaded at https://www.mdpi.com/article/10.3390/gels9040279/s1, Figure S1: High-resolution XPS spectra of PZ-PAA@Fe^3+^ bi-layer hydrogel; Figure S2: Equilibrium water contents of PZ hydrogel in response to NaCl concentrations; Figure S3: Equilibrium water contents of PAA@Fe^3+^ hydrogel in response to NaCl concentrations; Figure S4: Equilibrium water contents of PZ hydrogel in response to the environmental changes from cool alkali liquors at 25 °C to hot alkali liquors at 55 °C; Figure S5: Equilibrium water contents of PAA@Fe^3+^ hydrogel in response to the environmental changes from cool alkali liquors at 25 °C to hot alkali liquors at 55 °C; Figure S6: Comparison of bending behavior of the bi-layer hydrogel strips coordinating at different Fe^3+^ concentrations.
