# Supplementary material for: An Anisotropic Hydrogel by Programmable Ionic Crosslinking for Sequential Two-Stage Actuation under Single Stimulus
Source: Gels. 2023 Mar 29;9(4):279. doi: 10.3390/gels9040279 (PMC10137370; doi:10.3390/gels9040279)
Supplement: Supplementary file 1 [file gels-09-00279-s001.zip › gels-2283314-supplementary.pdf]

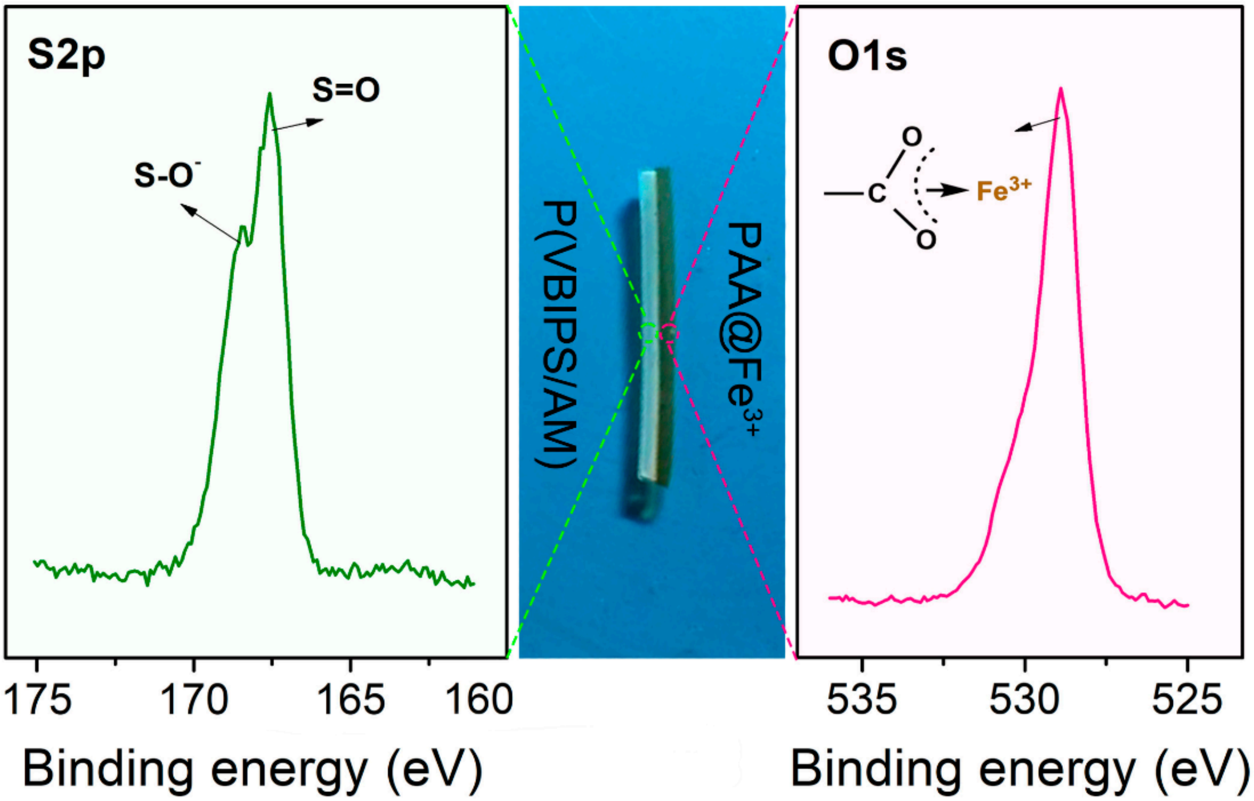

Figure S1. High-resolution XPS spectra of PZ-PAA@Fe^{3+} bilayer hydrogel.

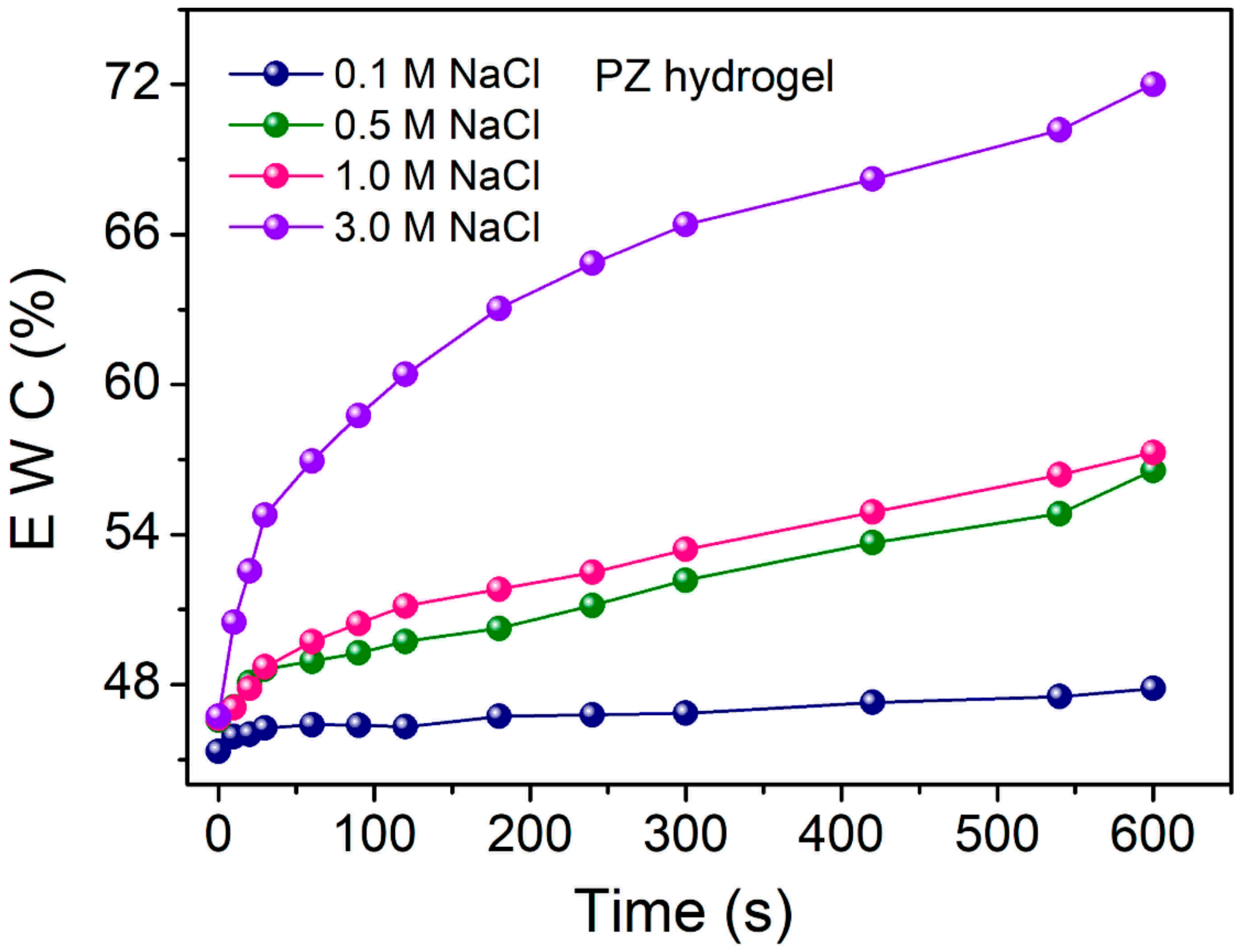

Figure S2. Equilibrium water contents of PZ hydrogel in response to NaCl concentrations.

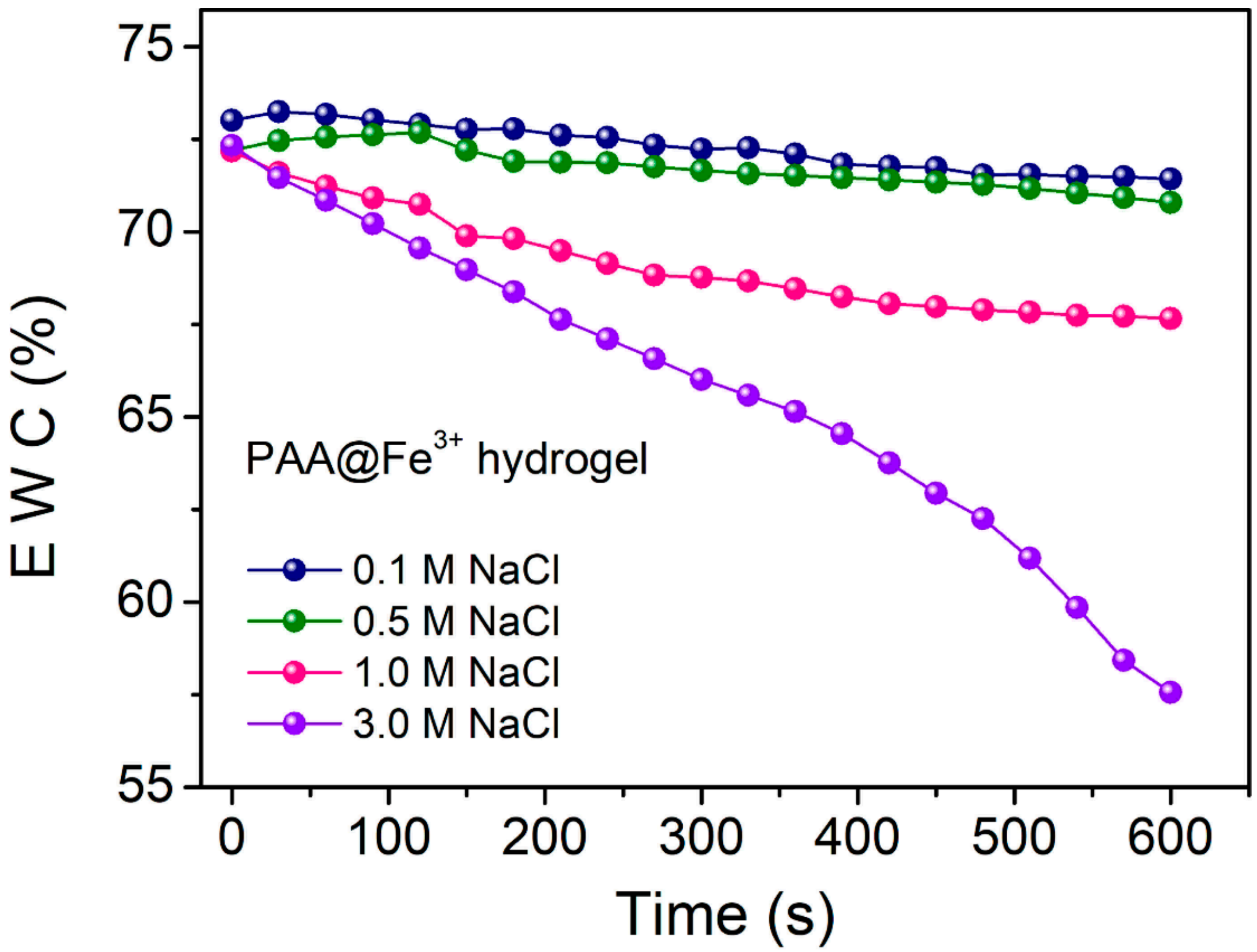

Figure S3. Equilibrium water contents of PAA@Fe^{3+} hydrogel in response to NaCl concentrations.

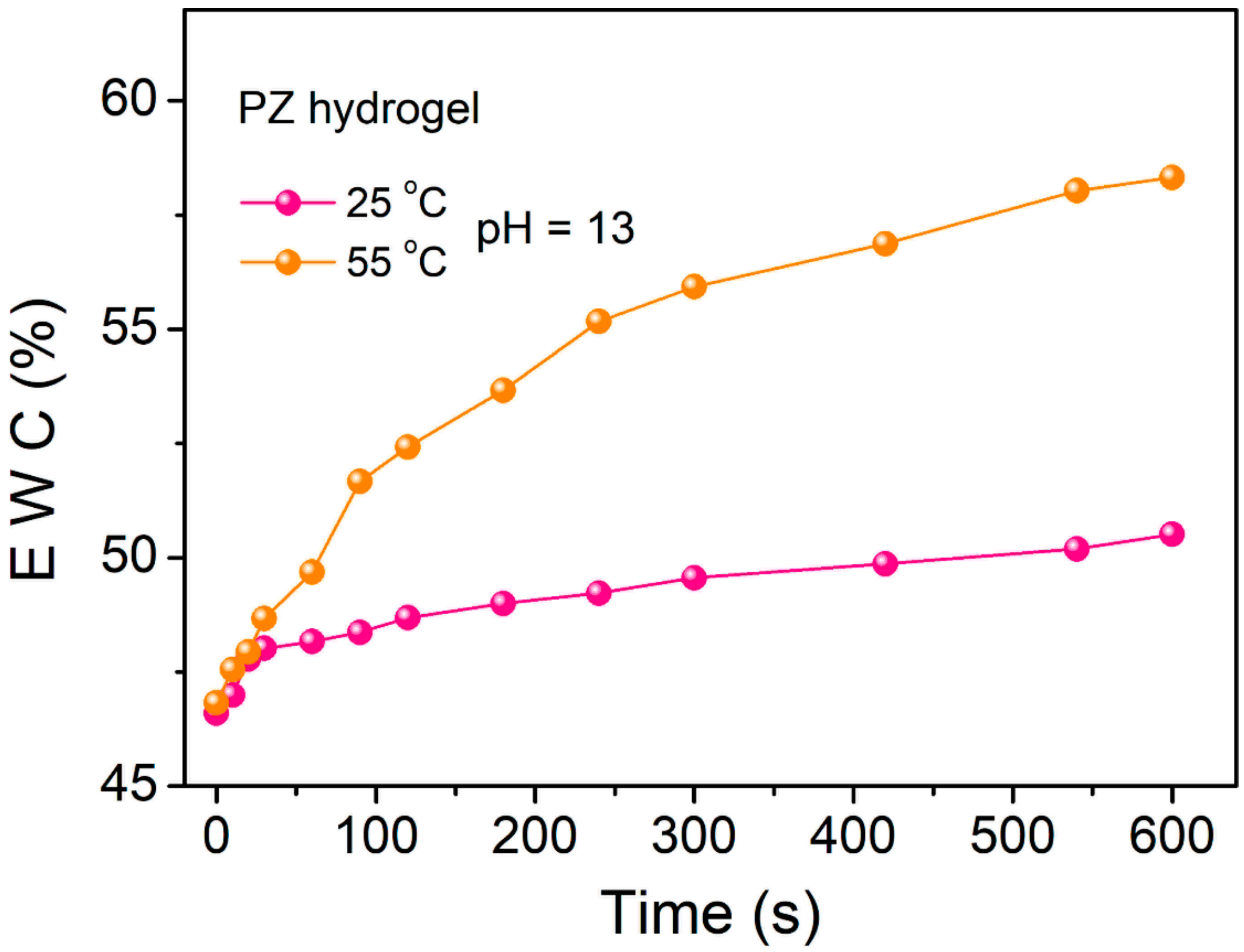

Figure S4. Equilibrium water contents of PZ hydrogel in response to the environmental changes from cool alkali liquors at 25 °C to hot alkali liquors at 55 °C.

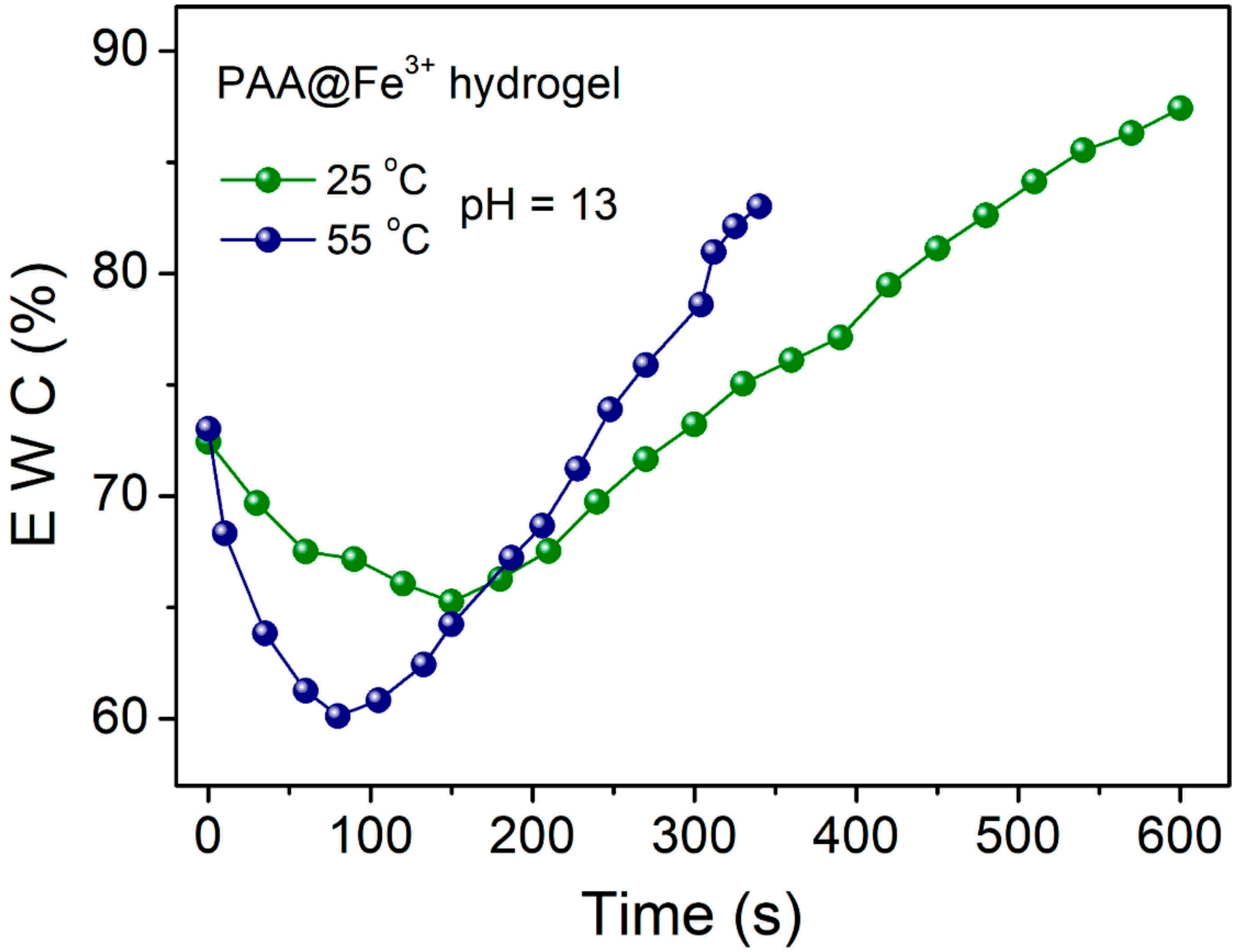

Figure S5. Equilibrium water contents of PAA@Fe^{3+} hydrogel in response to the environmental changes from cool alkali liquors at 25 °C to hot alkali liquors at 55 °C.

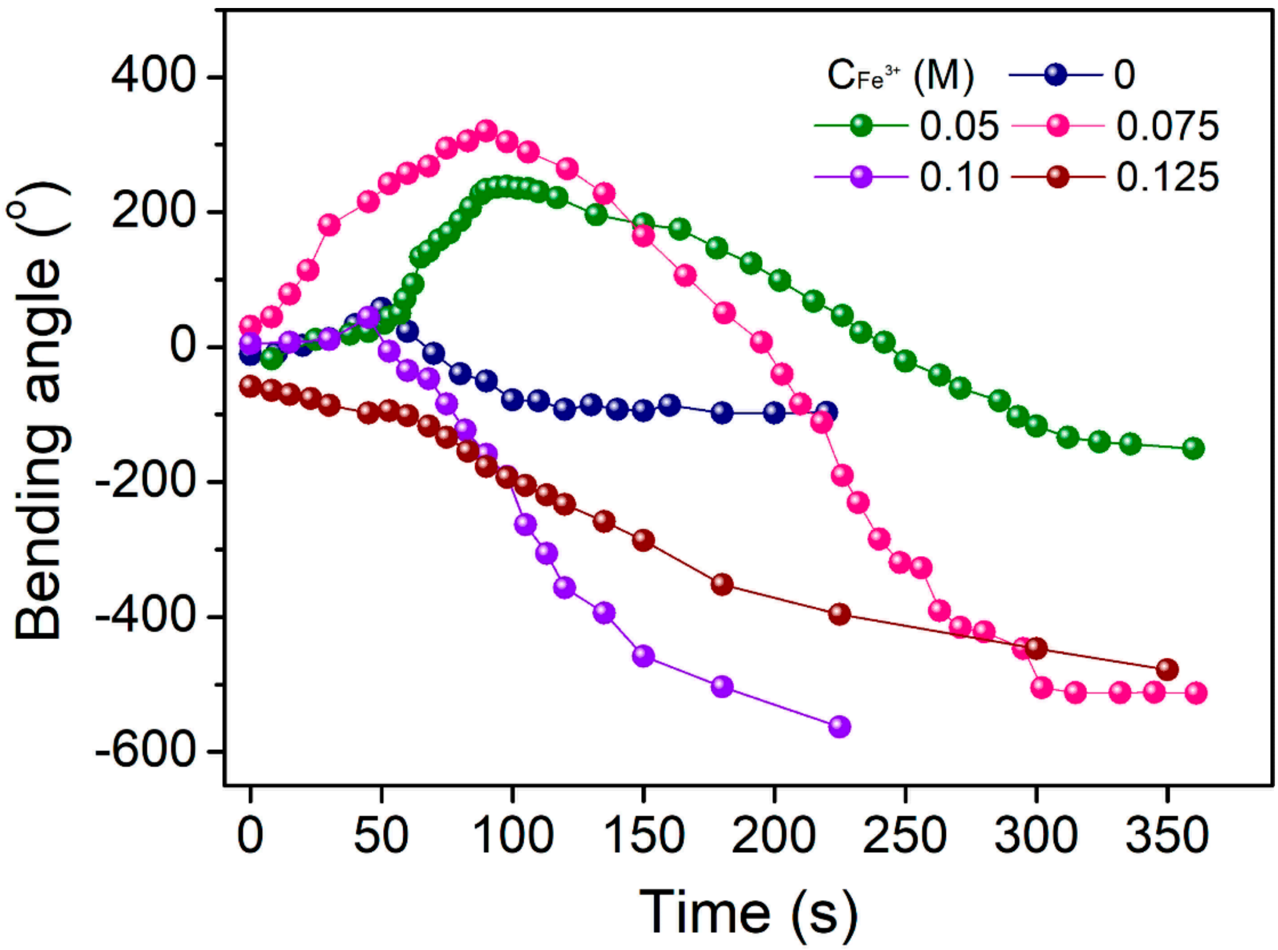

Figure S6. Comparison of bending behavior of the bilayer hydrogel strips coordinating with different Fe^{3+} concentrations.
